# Biological applications of the NanoSuit for electron imaging and X-microanalysis of insulating specimens

**DOI:** 10.1186/s42649-022-00073-2

**Published:** 2022-05-11

**Authors:** Ki Woo Kim

**Affiliations:** Department of Ecology and Environmental System, Kyungpook National University, Sangju, 37224 Republic of Korea

**Keywords:** NanoSuit, Scanning electron microscopy, Vacuum

## Abstract

Field emission scanning electron microscopy (FESEM) is an essential tool for observing surface details of specimens in a high vacuum. A series of specimen procedures precludes the observations of living organisms, resulting in artifacts. To overcome these problems, Takahiko Hariyama and his colleagues proposed the concept of the “nanosuit” later referred to as “NanoSuit”, describing a thin polymer layer placed on organisms to protect them in a high vacuum in 2013. The NanoSuit is formed rapidly by (i) electron beam irradiation, (ii) plasma irradiation, (iii) Tween 20 solution immersion, and (iv) surface shield enhancer (SSE) solution immersion. Without chemical fixation and metal coating, the NanoSuit-formed specimens allowed structural preservation and accurate element detection of insulating, wet specimens at high spatial resolution. NanoSuit-formed larvae were able to resume normal growth following FESEM observation. The method has been employed to observe unfixed and uncoated bacteria, multicellular organisms, and paraffin sections. These results suggest that the NanoSuit can be applied to prolong life in vacuo and overcome the limit of dead imaging of electron microscopy.

## Introduction

Scanning electron microscopy (SEM) is widely used to observe fine details of organisms in high vacuums. In particular, field emission SEM (FESEM) needs a higher level of vacuum (10^− 3^ to 10^− 7^ Pa) for high-resolution imaging than conventional SEM (Kasahara et al., 2019). The effects of high vacuums on organisms include rapid evaporation of water across their surface layer as well as the collapse and death of organisms (Takaku et al. 2013). Only anhydrobiotic organisms that can survive nearly complete desiccation, such as tardigrades commonly known as water bears, have the potential to survive space vacuums (10^− 4^ to 10^− 15^ Pa) (Jönsson et al. 2008). Most multicellular organisms can thrive under atmospheric or higher-than-atmospheric (deep-sea) pressures (Takaku et al. 2013).

For SEM imaging, most organisms are subject to specimen preparations including chemical fixation, dehydration, critical point drying, and conductive coating (Kim 2020; Sun et al. 2021). These harsh procedures preclude the observation of living organisms in high vacuums using FESEM, leading to unwanted artifacts. It has been thought that it is impossible to observe living organisms using electron microscopy (Hariyama et al. 2020). In order to overcome these problems, electron imaging under reduced vacuum is employed with lower magnification and resolution (Kim 2013). However, recent findings have provided some evidence that living multicellular organisms can be observed using FESEM .

### Nanosuit formation by electron beam irradiation

First proposed by Takahiko Hariyama and his colleagues in 2013, the concept of the “nanosuit” was developed to observe live organisms in high vacuums using FESEM (Takaku et al. 2013). They found that a surface modification by electron beam irradiation produced the nanosuit, a thin extra layer on the specimen. Since the layer was flexible and dense enough to keep the living organism’s gases and liquids from evaporating, it works like a miniature spacesuit, and was designated as “nanosuit” (Takaku et al. 2017). A low magnification (20–30 X) electron beam can be used to irradiate the entire surface of the specimen (Takaku et al. 2020).

**Fig. 1:**
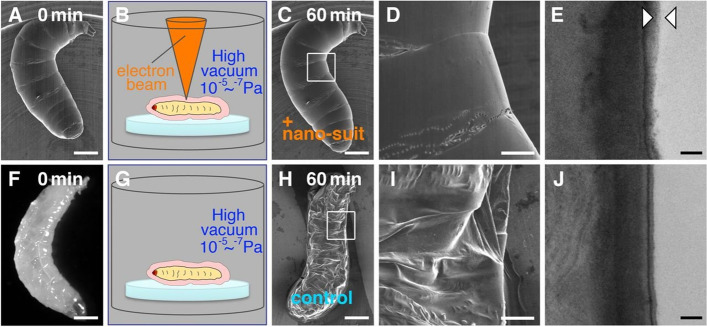
Fruit fly *Drosophila melanogaster* larvae. **A**–**E** Larva irradiated with electron beam for 60 min. **A**, **C**, and **D** SEM images. **B** Schematic drawing. **E** TEM image. Arrowhead = extra surface layer. **F**–**J** Larva not irradiated with electron beam for 60 min. **F** Optical micrograph. **G** Schematic drawing. **H** and **I** SEM images. **J** TEM image. Bars = 0.3 mm (**A**, **C**, **F**, and **H**), 0.1 mm (**D** and **I**), and 0.2 μm (**E** and **J**). From Takaku et al. 2013 with permission from the publisher

The nanosuit rendered the fruit fly *Drosophila melanogaster* larvae tolerant to a high vacuum (10^− 5^–10^− 7^ Pa) at 5 kV among other organisms belonging to various taxa (Fig. 1A–D). Transmission electron microscopy (TEM) showed an extra thin layer (50–100 nm thick) on the surface (Fig. 1E). In contrast, larvae without electron beam irradiation showed severe distortions (Fig. 1F–I). What was not accounted for is the absence of exposure in the underside of the specimens mounted on stubs. The unexposed surface would leave the specimens vulnerable to water loss and thus collapse. No extra surface layer was observed in the non-irradiated larvae (Fig. 1J). Therefore, the thin polymerized layer caused by electron beam irradiation was hypothesized to act as a barrier to protect the organism in vacuums.

### Nanosuit formation by plasma irradiation

**Fig. 2:**
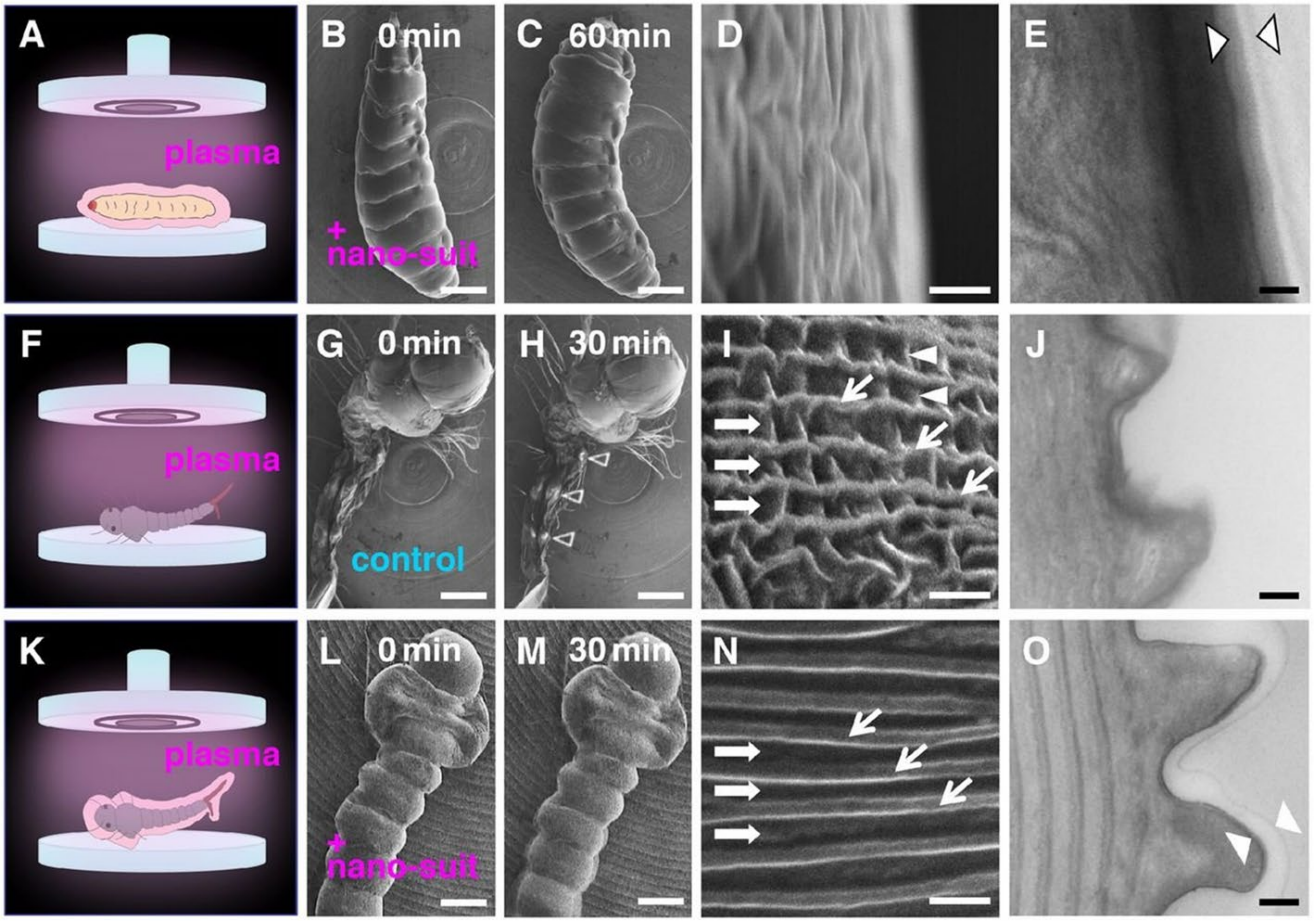
Insect larvae. **A**–**E** *Drosophila* larva irradiated with plasma for 3 min observed using SEM for 60 min. **A** Schematic drawing. **B** to **D** SEM images. **E** TEM image. Arrowhead = extra surface layer. **F**–**J** Mosquito *Culex pipiens molestus* larva (no extracellular substances) irradiated with plasma for 3 min observed using SEM for 30 min. **F** Schematic drawing. **G**–**I** SEM images. Arrowhead (**H** and **I**) = area of electrostatic charging. Arrows (**I**) = furrows with wrinkles. **J** TEM image. **K**–**O** Mosquito larva irradiated with electron beam and treated with plasma-irradiated Tween 20. **K** Schematic drawing. **L**–**N** SEM images. Arrows (N) = furrows without wrinkles. **O** TEM image. Arrowhead = extra surface layer. Bars = 0.3 mm (**B**, **C**, **G**, **H**, **L**, and **M**), 1 μm (**D**, **I**, and **N**), and 0.2 μm (**E**, **J**, and **O**). From Takaku et al. 2013 with permission from the publisher

To test the hypothesis, the authors tried to construct this nanosuit on different insect larvae by plasma irradiation. The plasma-irradiated polymerization has been used to produce ultrathin polymer-like layers with a defined and regular structure (Friedrich 2011). Specimens were irradiated with plasma inside an ion-sputtering device without a metal emitter for 3 min and observed using FESEM for 60 min (Takaku et al. 2013). The plasma-irradiated larvae of *Drosophila* having extracellular substances (ECS) were similar in structure to those irradiated by electron beams at 5 kV (Fig. 2A–E). In contrast, larvae of *Culex pipiens molestus* having no ECS shrank quickly (Fig. 2F–J), which was similar to those of the untreated control group in Fig. 1.

Based on the chemical analysis of natural ECS, the polyoxyethylene (20) sorbitan monolaurate (Tween 20) had been selected as a biomimetic agent for its nontoxic compound having amphiphilic molecules (Takaku et al. 2013). The mosquito larvae were immersed in 1% (v/v) Tween 20 solution dissolved in distilled water for 1 min, irradiated with plasma, and observed using FESEM showed similar structures to those irradiated by electron beams (Fig. 2K–O). TEM analysis of plasma-irradiated Tween 20 film revealed an electron density gradient from the irradiated to the unirradiated surface (Suzuki et al. 2013). Taken together, the artificial ECS, Tween 20 film following plasma irradiation, was crucial to the specimen protection against high-vacuum electron imaging.

### From nanosuit to NanoSuit^®^

Both the natural and artificial ECS were referred to as the “nanosuit” in the early work published by the group (Takaku et al. 2013). Later, Takahiko Hariyama and his colleagues coined the term “NanoSuit” to describe “a thin vacuum-proof polymer layer on the specimen” that keeps the organism alive in high vacuums (Ohta et al. 2014). The NanoSuit is formed instantly by (i) electron beam irradiation, (ii) plasma irradiation, and (iii) Tween 20 solution (Table 1). They could observe the details of living insect specimens at high magnifications (500,000 X) with a simpler, less-time-consuming procedure through the surface-shield-effect caused by the NanoSuit (Ohta et al. 2014). The NanoSuit was shown to prolong the charge-free conditions and increase survival time under the high vacuum (Takaku et al. 2015).

**Table 1:** Comparison of NanoSuit versions for live imaging and elemental analysis

NanoSuit^a^	Modified NanoSuit^b^
(i) Electron beam irradiation (EBI)	Immersion in surface shield enhancer solution (glycerin and electrolytes) plus EBI
(ii) Plasma irradiation (PI)	
(iii) Immersion in 1% Tween 20 aqueous solution plus EBI or PI	

^a^Three methods for producing NanoSuit depending on organisms from Takaku et al. 2013

^b^from Takaku et al. 2017

Based on their invention, three types of NanoSuit^®^ aqueous solutions are currently commercially available from an electron microscopy chemical supplier. Three types of solutions are for living tissues/materials (small animals, plants, and food), corelative light and electron microscopy (paraffin-fixed sections), and cells (bacteria, liposomes, and viruses), respectively. Beyond biological specimens, the simple surface modification can be applied to food protection through the pinhole-free film using the NanoSuit method (Hariyama et al. 2020).

### Modified NanoSuit: SSE solution

There were instances where the Tween 20-based NanoSuit was not effective at imaging excised tissues and cultured cells. A modified NanoSuit was proposed by the same research group in 2017 (Table 1) to overcome this problem. They developed a surface shield enhancer (SSE) solution, which was a mixture of glycerin and electrolytes, conceived due to the hygroscopic nature of glycerin. The SSE solution was made of sucrose, fructose, and sodium chloride in distilled water, to which citric acid and sodium glutamate (pH 7.4) were added (Takaku et al. 2017). The resulting solution was mixed with glycerin at a ratio of 1:2.

**Fig. 3:**
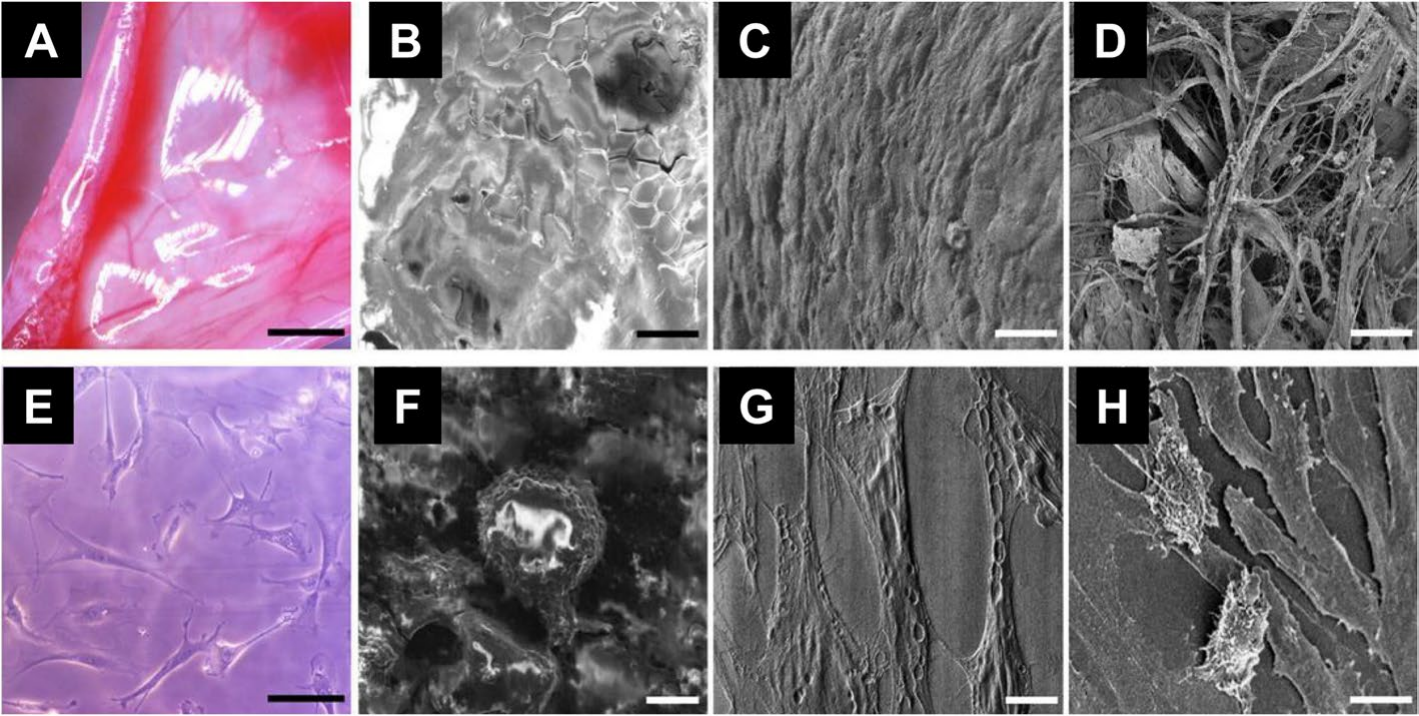
Mouse peritoneum and embryonic fibroblast cells. **A** and **E** Light microscopy images. **B** and **F** Untreated specimens. **C** and **G** Specimens treated with the surface shield enhancer solution. **D** and **H** Specimens conventionally processed. Bar(s) = 5 mm (**A**) and 10 μm (**B**–**D** and **F**–**H**). From Takaku et al. 2017 with permission from the publisher

Excised mouse peritonea (Fig. 3A) and mouse embryonic fibroblast cells (Fig. 3E) were immersed in the SSE solution for 1 min, blot dried, and observed using FESEM at 1 kV. They were also processed using a conventional specimen protocol. Untreated, control specimens became shrunken and showed charging (Fig. 3B and F). However, the SSE solution-treated specimens maintained their shape without charging (Fig. 3C and G), which was different from that of the conventionally processed specimens (Fig. 3D and H). The SSE solution-based layer (less than 10 nm thick) was thinner than the Tween 20 solution-based one (50 to 200 nm thick) in TEM images (Takaku et al. 2017). Accordingly, the SSE solution-based NanoSuit may provide higher resolution surface images than the previous NanoSuit methods.

### Uses of the NanoSuit method

#### Organisms

##### Insects

Since early NanoSuit investigations, insects have been rigorously used to observe the differences in shape and weight according to different SEM specimen preparations. Since only some insect forms, including dipteran larvae possess ECS on their surface, the NanoSuit inventors tried to produce artificial ECS on other insects. The NanoSuit-formed specimens actively moved around for 60 min during the FESEM observation (Takaku et al. 2013). After FESEM works, the NanoSuit-formed larvae developed into normal adults. A combination of chemical fixation and NanoSuit method was successful at imaging the first instar nymphs (Mashimo and Machida 2017). The NanoSuit was useful for insect embryology because the same embryos could be reused for routine histological analyses after SEM observation (Fujita et al. 2016).

##### Plants

**Fig. 4:**
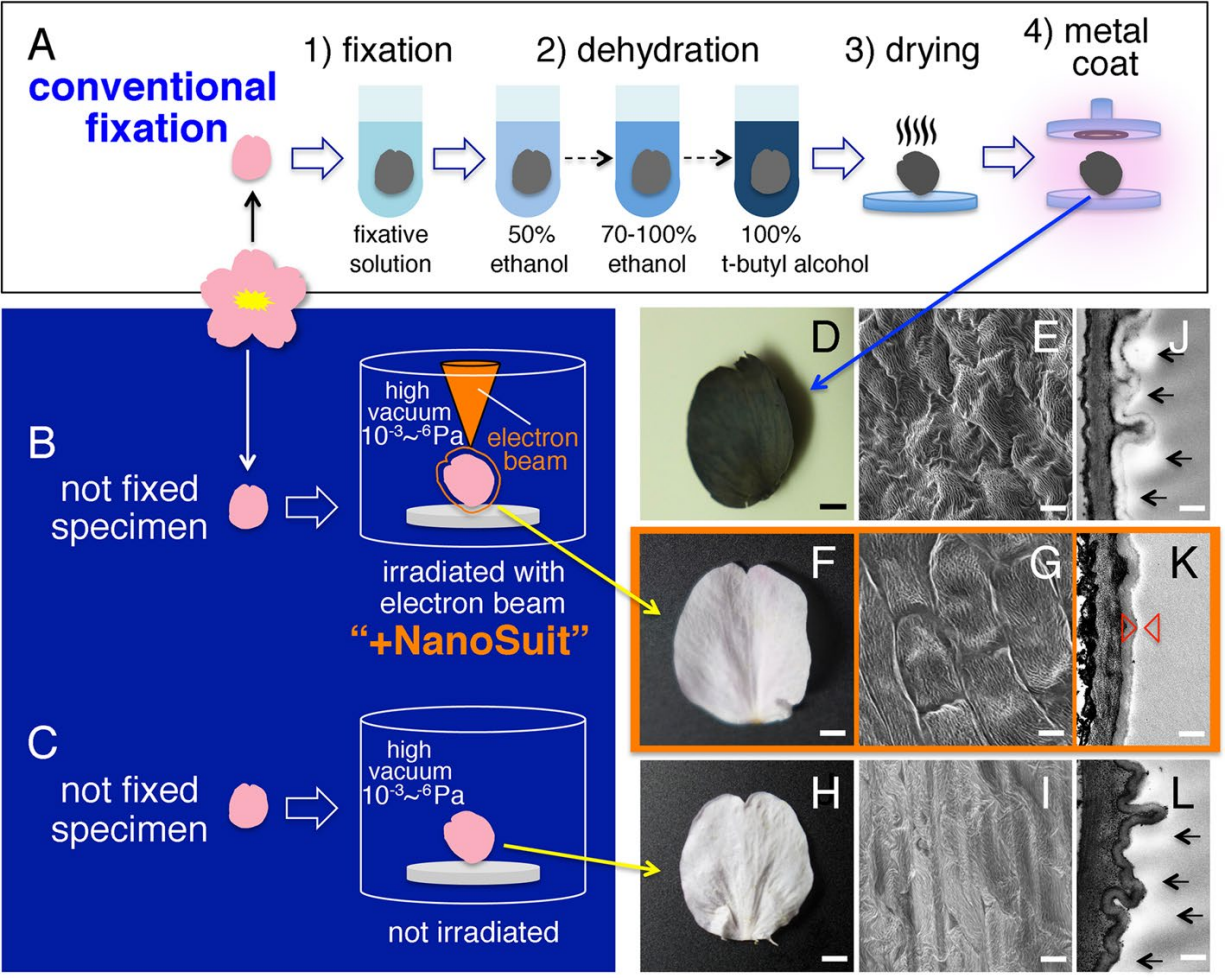
Cherry blossom petals. **A**–**C** Schematic drawings of a conventional fixation protocol, a NanoSuit method with beam irradiation and no fixation, and a control with neither beam irradiation nor fixation. **D**, **E**, and **J** Photograph, SEM image, and TEM image of the conventionally-processed petal. Arrow = surface material. **F**, **G**, and **K** Photograph, SEM image, and TEM image of the NanoSuit-formed petal. Arrowhead = polymerized surface layer. **H**, **I**, and **L** Photograph, SEM image, and TEM image of the control petal. Bars = 2 mm (**D**, **F**, and **H**), 10 μm (**E**, **G**, and **I**), 300 nm (**J**, **K**, and **L**). From Takehara et al. 2020 with permission from the publisher

The NanoSuit method has been mainly utilized to observe animal specimens. Furthermore, it can be applied to other organisms including plants (Haque and Matsubara 2018; Takehara et al. 2018; Takaku et al. 2020). For example, petals of cherry blossoms (*Prunus* spp.) underwent the conventional protocol with freeze-drying using t-butyl alcohol (Fig. 4A), the NanoSuit method (Fig. 4B) and no treatment (Fig. 4C) at 1 kV (Takehara et al. 2020). The conventionally processed petal showed shrinkage and an extra layer of material on the surface (Fig. 4D, E, and J). The NanoSuit-formed petal maintained a nearly intact surface and an extra thin layer on the surface (Fig. 4F, G, and K). The thin layer was assumed to be polymerized by electron beam irradiation, keeping the petal hydrous in the SEM chamber. Conversely, the untreated petal was shrunken and had an extra layer on the surface (Fig. 4H, I, and L). The NanoSuit took a few minutes and maintained the hydrous specimen with a thin polymerized layer on the specimen surface.

The cherry blossom petals were treated with chloroform and directly observed using FESEM (Takehara et al. 2018). While the electron beam-irradiated specimens were intact, the chloroform-treated specimens showed shrinkage and collapse. These results suggest that the NanoSuit for plants may be closely associated with epicuticular waxes easily extracted with chloroform. Furthermore, electron-translucent layers or NanoSuit on the petal surface in TEM images were similar to those in the cross profile of leaf epicuticular waxes (Kim 2008).

Thirteen plant species belonging to different taxa were selected, irradiated with an electron beam to form the NanoSuit, and observed using FESEM. The images were recorded at magnifications from 100 X to 10,000 X to provide open image datasets that can be freely viewed (Takehara et al. 2020).

Plant specimens for X-ray microanalysis of soluble salts are commonly air dried and coated with gold. Strawberry petioles and roots under salinity stress were immersed in 1% Tween 20 solution, plasma irradiated, and observed using SEM (Haque and Matsubara 2018). The uncoated plant parts maintained structural details and provided correct data for sodium (Na) localization.

##### Fungi

**Fig. 5:**
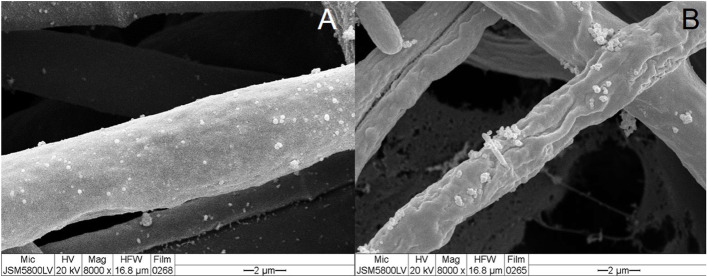
SEM images of *Stagonosporopsis cucurbitacearum* hyphae. **A** Hyphae treated with crude metabolites of muskmelon seedlings **B** Hyphae treated with crude metabolites of muskmelon seedlings inoculated with *Trichoderma asperelloides*. Note the abnormal cell walls (arrows). From Ruangwong et al. 2021 with permission from the publisher

A filamentous fungus *Trichoderma asperelloides* was grown on the nylon membrane (Ruangwong et al. 2021). The membrane was immersed in 0.1% (v/v) Tween 20 solution and blotted with a filter paper. The specimen was then irradiated by plasma, sputter-coated with gold, and observed using SEM at 20 kV. Hyphae of a muskmelon fungal pathogen *Stagonosporopsis cucurbitacearum* treated with crude metabolites from muskmelon seedlings showed typical cell walls (Fig. 5A). However, crude metabolites from muskmelon seedlings inoculated with *T. asperelloides* showed abnormal cell walls (Fig. 5B). The protocol was different from the original version in that the Tween-treated specimens were gold-coated before SEM observation.

##### Bacteria

An intense study of prokaryotes using the NanoSuit method has not occurred. Meanwhile, bacteria were seen in paraffin sections of human adrenal tissues (Kawasaki et al. 2020). The NanoSuit was formed by applying the SSE solution to the section surface for 1 min. Rod-shaped bacteria were clearly visualized in the section and later identified as *Aeromonas hydrophila*.

##### Sharks

Sharkskin surfaces have been employed as an effective biomimetic design template for flow control and drag reduction (Fu et al. 2017). As an integrated study for three-dimensional structural analysis, five specimens of fresh sharkskin were immersed in 1% Tween 20 solution in distilled water for 1 min (Miyazaki et al. 2018). The specimens were then observed using FESEM at 1 kV. Denticles had grooves arranged in a hound’s-tooth-check pattern. No apparent differences were found in denticle structures among the species. A surface shielding was assumed to be formed on the specimen surface by electron beam irradiation.

### Cancer cells

The modified NanoSuit method was applied to cancer research. Colorectal cancer tissues and their adjacent normal mucosa were immersed in the SSE solution for 1 min, blot dried, and observed using FESEM (Kikuchi et al. 2017). Conventionally processed specimens showed inevitable structural damages. The SSE solution-treated colorectal cancer lesion possessed a relatively amorphous surface and fiber-like structure, distinguished from non-cancerous mucosa in colon tissues. Similar results were shown in surgical explants of human stomach wall including areas of cancerous tissue (Takaku et al. 2017). SSE solution-treated specimens appeared intact, whereas conventionally fixed and processed ones showed structural damages.

### Immunohistochemistry

**Fig. 6:**
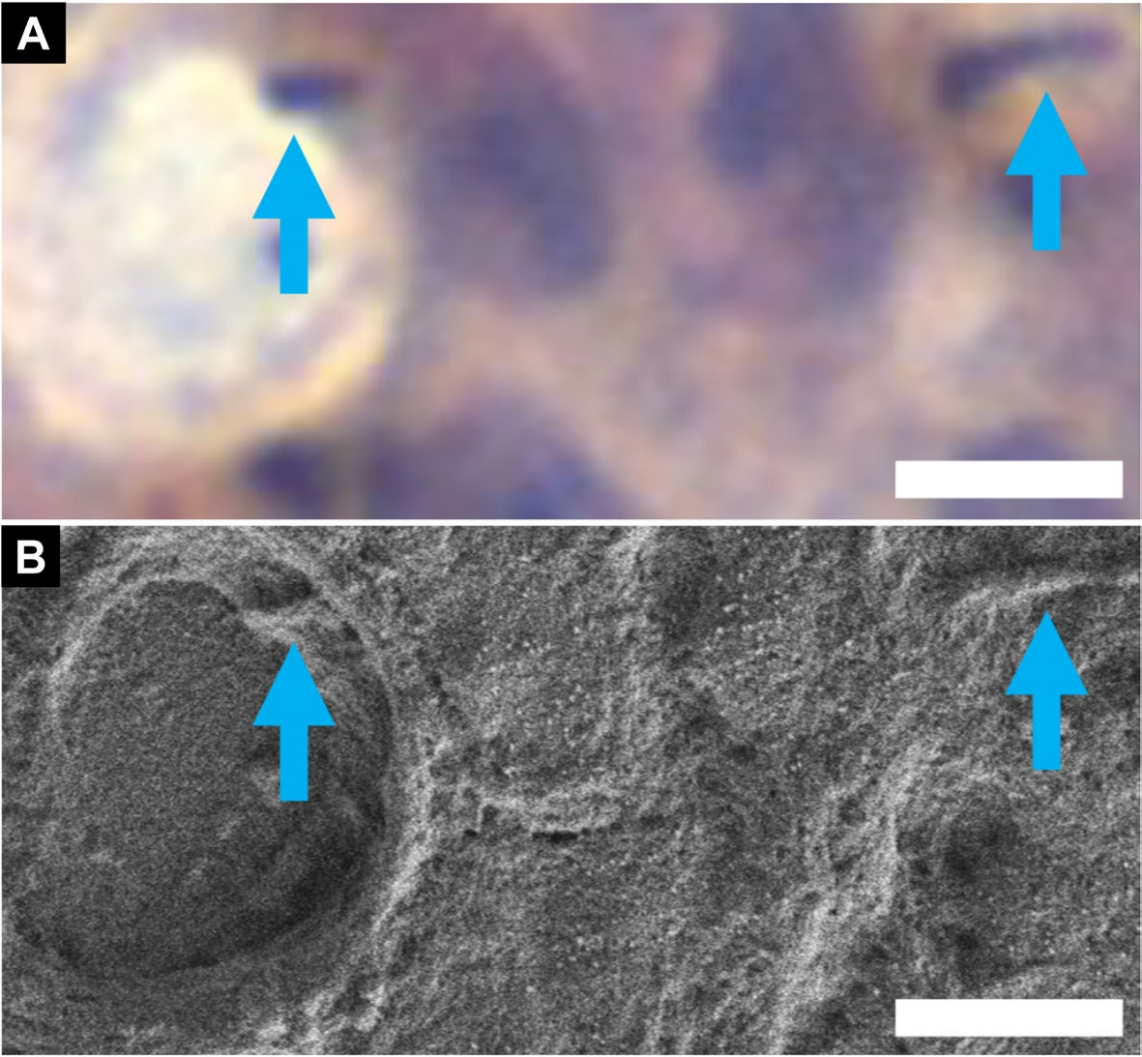
Immunohistochemistry. **A** Light microscopy image of primary cilia (arrows) in human salivary gland tumors after ARL 13B immunohistochemistry. **B** Corresponding SEM image after the NanoSuit treatment. Bars = 5 μm. From Shinmura et al. 2021 with permission from the publisher

Primary cilia (PC) are long, thin, non-motile, and antenna-like structures protruding from the apical surface of almost all cell types, most commonly from epithelial cells (Wheway et al. 2018). In order to provide a link between PC and salivary gland tumors, paraffin sections were used for immunohistochemistry (Fig. 6A) (Shinmura et al. 2021). The sections were rehydrated with the SSE solution and observed using FESEM (Fig. 6B).

### Correlative light and electron microscopy

**Fig. 7:**
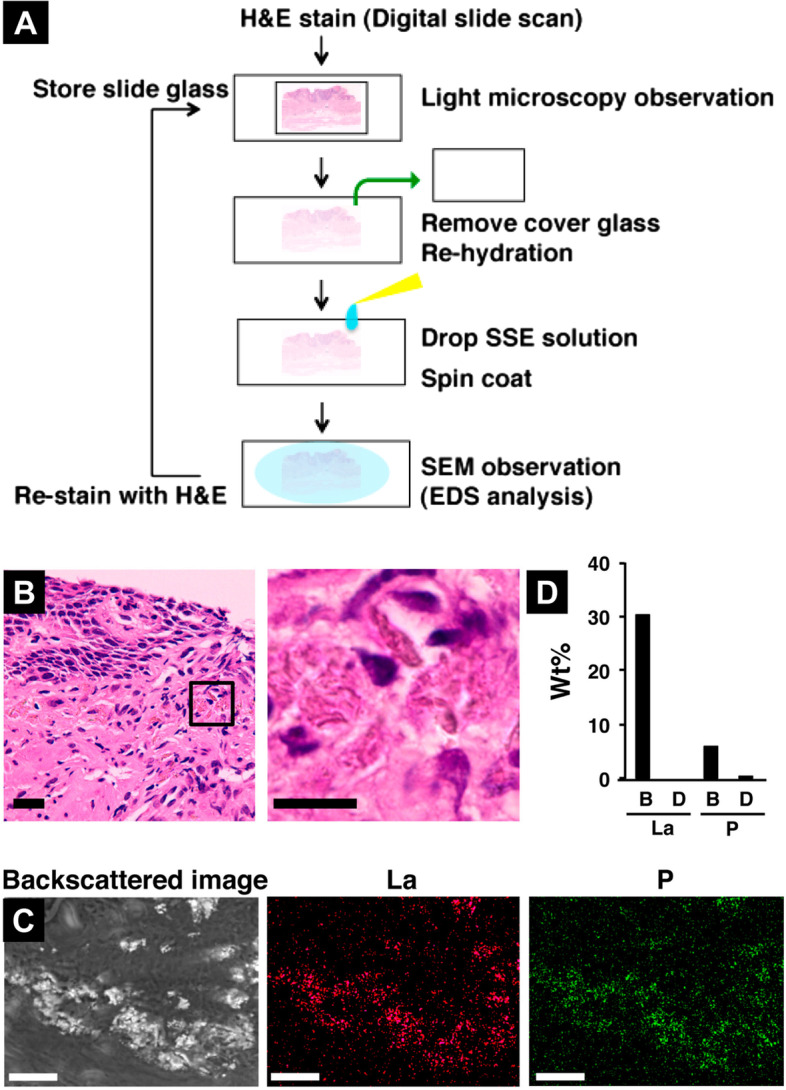
Correlative microscopy. **A** Schematic drawing of a correlative electron and light microscopy workflow with SEM observation and X-ray microanalysis. **B** Left: H&E-stained esophageal mucosa containing brown pigment deposition. Bar = 20 μm. Right: Magnified view of a square in the Left. Bar = 10 μm. **C** SEM observation and X-ray microanalysis. Left: Backscattered electron image. Middle: Lanthanum (La) map. Right: Phosphorous (P) map. Bars = 25 μm. **D** Weight (Wt) percent of La and P at the bright area (**B**) and the other dimmer area (**D**) of the Left panel of (**C**). From Shinmura et al. 2019 with permission from the publisher

A correlative light and electron microscopy (CLEM) method can be coupled with the NanoSuit method. For an in-depth diagnosis, it is necessary to combine a conventional hematoxylin and eosin (H&E) staining workflow with SEM observation and X-ray microanalysis. After removing the cover glass with xylene, the slide section was rehydrated with a drop of the SSE solution in distilled water (Fig. 7A) (Shinmura et al. 2019). H&E staining showed brown pigment deposition as possible regions of lanthanum (La) and phosphorus (P) deposition (Fig. 7B). Backscattered electron imaging revealed white regions containing La and P at 15 kV (Fig. 7C and D). These results suggest that the NanoSuit-CLEM could become a powerful tool for clinical practice in pathological diagnosis.

### Liquid marbles

**Fig. 8:**
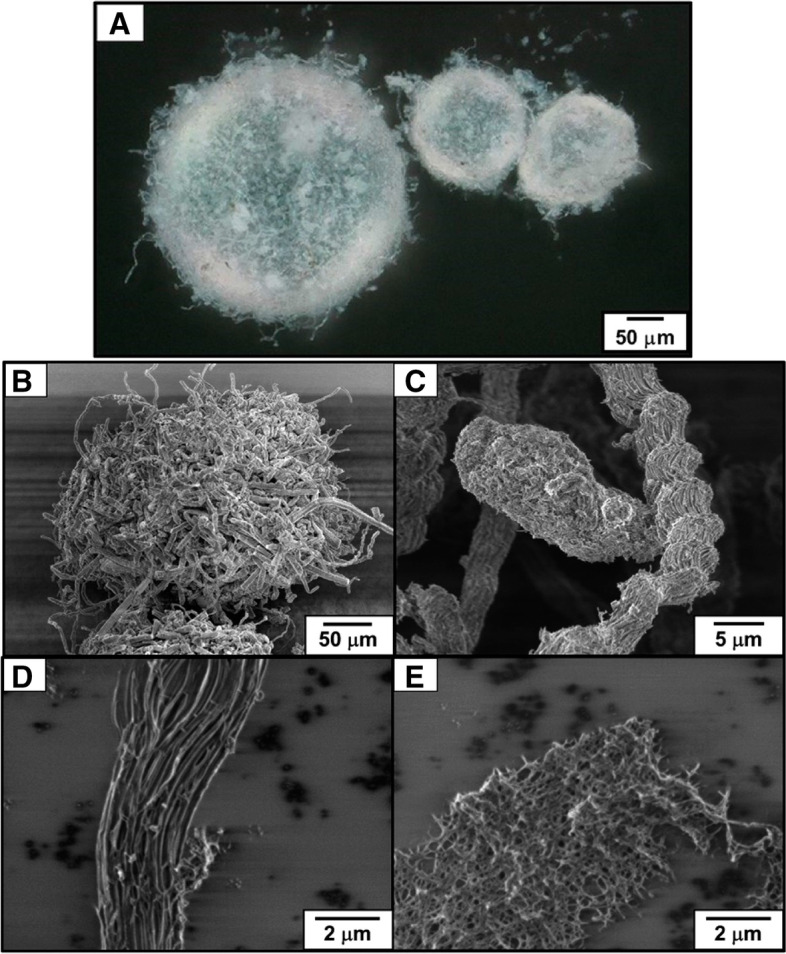
Liquid marbles (LMs). **A** Stereomicroscopy image of LMs fabricated by the gall-forming aphid *Eriosoma moriokense*. **B**–**E** FESEM images of the LMs treated with the NanoSuit method. From Kasahara et al. 2019 with permission from the publisher

Liquid marbles (LMs) are non-stick droplets wrapped by micro- or nanometer-sized hydrophobic solid objects (Bormashenko 2011). In nature, a gall-forming aphid *Eriosoma moriokense* forms LMs by wrapping its honeydew with wax, removing LMs out of the galls (Kasahara et al. 2019). Stereomicroscopy images revealed a near-spherical morphology of LMs (Fig. 8A). Without any preparations such as fixation and metal coating, the LMs were introduced into a FESEM. The LMs had protruded fiber-shaped wax (Fig. 8B), a helix-like form (Fig. 8C), and fiber- and net-shaped wax (Fig. 8D and E). A thin layer was considered to be formed by electron beam irradiation.

### Nanoparticle assay

**Fig. 9:**
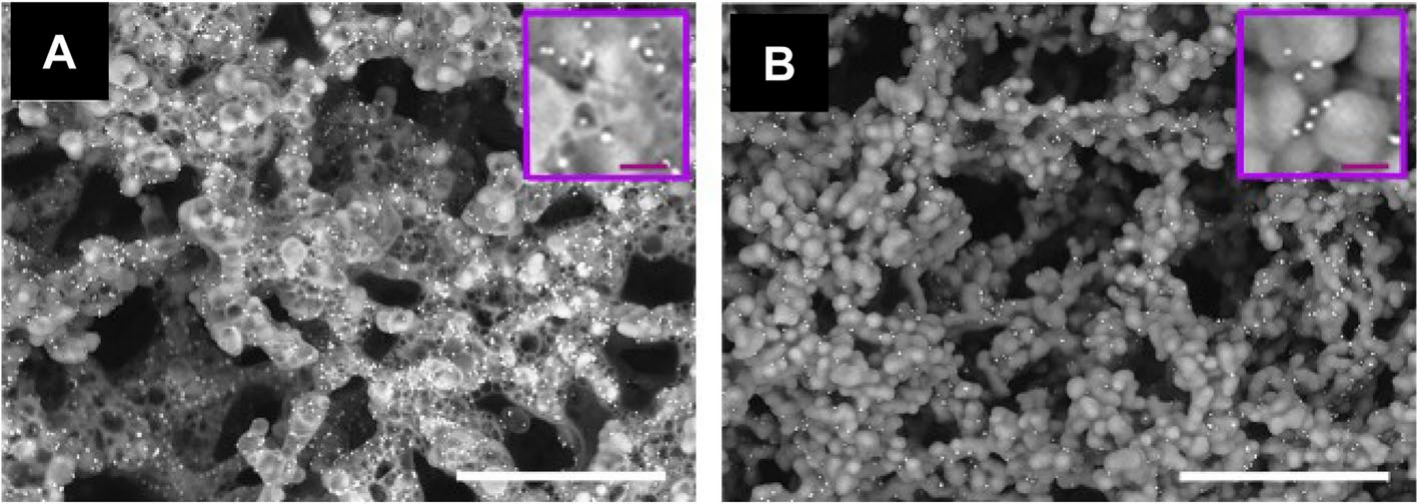
Backscattered electron images of gold/platinum particle-labeled immunocomplexes on cellulose membrane. **A** Without the NanoSuit treatment. **B** With the NanoSuit treatment. Bars = 15 μm. Insets = magnified views (bars = 600 nm). From Kawasaki et al. 2021 with permission from the publisher

Diagnostic tests should be equipped with high sensitivity and rapid performance for practical use. Although nanoparticle-based platforms have been used in biomedical and environmental sciences, direct observation of nanoparticles using electron microscopy is limited in diagnostic tests (Agasti et al. 2010). Immunocomplexes with gold/platinum nanoparticles were treated with a modified NanoSuit solution with Tween 20-based components (25 μl., twice) and observed using a desktop SEM at 10 or 15 kV (Kawasaki et al. 2021). Backscattered electron imaging revealed that cellulose swelling was apparent without the NanoSuit treatment (Fig. 9A). However, the cellulose was not swollen with the NanoSuit treatment (Fig. 9B). This workflow could offer high sensitivity and simplicity by direct particle observation.

### Droplet spotter system

**Fig. 10:**
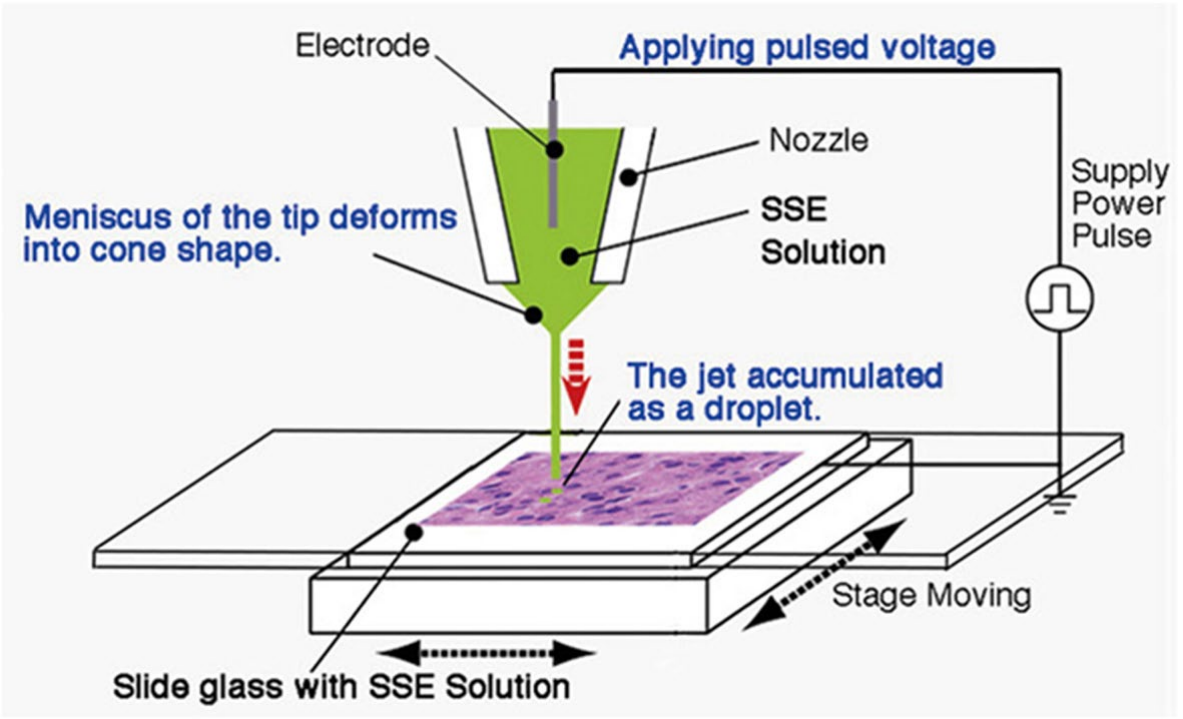
Droplet spotter system. Minute droplets of undiluted surface shield enhancer (SSE) solution are applied at multiple locations with the computer-controlled system. From Kawasaki et al. 2020 with permission from the publisher

The NanoSuit method can be performed using an ECS-mimicking substance, also called SSE solution, which polymerizes to form a shield through an electron beam or plasma irradiation. This work can be facilitated through the use of a computer-controlled droplet spotter system (Fig. 10) (Kawasaki et al. 2020). If a voltage is applied between the SSE solution and a specimen, a minute volume (pl to fl) of the solution is ejected from the nozzle and forms droplets at precise locations. Spotted slides are introduced to SEM, and the NanoSuit can be formed as a thin and vacuum-proof membrane.

## Conclusions

Conventional SEM specimen procedures comprise harsh steps, leading to organismal death and artifacts. To surmount these problems, Takahiko Hariyama and his colleagues proposed the concept of “nanosuit”, later referred to as “NanoSuit”, describing a thin polymer layer on the organisms for protecting them in high vacuum. The NanoSuit is formed immediately by (i) electron beam irradiation, (ii) plasma irradiation, (iii) Tween 20 solution immersion, and (iv) SSE solution immersion. The method has been applied to various organisms, paraffin slide sections, and biomedical  specimen purposes. The NanoSuit-formed specimens allowed structural preservation and accurate element detection of insulating, wet specimens at high spatial resolution (Takaku et al. 2020).

Other solution-based methods, such as ionic liquids and BEL-1, have been used for SEM specimen procedures (Kuwabata et al. 2006; Takahashi et al. 2018). Despite the wide use of ionic liquids for SEM imaging, we have to be cautious about the toxicity of ionic liquids to organisms (Gonçalves et al. 2021). Given the resumed normal growth of NanoSuit-formed organisms following FESEM imaging, the NanoSuit treatment appears to be confined to the cell surface. In contrast, a room temperature ionic liquid was assumed to penetrate red blood cells (Mutoh et al. 2015). Further works await the elucidation of mechanisms underlying the NanoSuit-induced structural preservation and electrical conductivity. Beyond the paradigm of a more straightforward SEM method, the NanoSuit could be conceived as a means for prolonging life in vacuo as well as overcoming the limit of dead imaging of electron microscopy.

## Data Availability

Data and materials available on request.

## Abbreviations

ECS: Extracellular substances
FESEM: Field emission scanning electron microscopy
H&E: Hematoxylin and eosin
SEM: Scanning electron microscopy
SSE: Surface shield enhancer
TEM: Transmission electron microscopy
